# Does obesity Kuznets curve exist in developing economies? Evidence from 38 African countries based on heterogeneous panel data analysis on income-level classification

**DOI:** 10.3389/fpubh.2023.1200555

**Published:** 2023-11-02

**Authors:** Hao Chen, Samuel Atingabili, Isaac Adjei Mensah, Akoto Yaw Omari-Sasu, Evelyn Agba Tackie, Francisca Arboh, Bertha Ada Danso

**Affiliations:** ^1^School of Management, Jiangsu University, Zhenjiang, China; ^2^Department of Statistics and Actuarial Science, Kwame Nkrumah University of Science, Kumasi, Ghana; ^3^Institute of Applied Systems Analysis (IASA), School of Mathematical Science, Jiangsu University, Zhenjiang, China; ^4^Hospitality Management Department, Takoradi Technical University, Takoradi, Ghana

**Keywords:** obesity, economic growth, Africa, obesity Kuznets curve, urbanization, trade, unemployment

## Abstract

**Introduction:**

The global pandemic disease known as the obesity epidemic has spread throughout the planet. Particularly, Africa is facing a growing problem of obesity, and the trend is rising. This is a result of a ticking time bomb. Given the claim that multiple socio-economic factors significantly affect the diversity in obesity rates between nations, economic development can be seen as a key contributor to this variation.

**Methods:**

Relying on the aforementioned avowal, this extant research examines the relationship between obesity and economic growth using urbanization, trade openness, and unemployment as intermittent variables within the Obesity Kuznets Curve (OKC) framework. Using panel data from 1990 to 2020, a panel of 38 African countries subdivided into income levels (Low income, Lower-middle income, and Upper-middle income) were analyzed. With the presence of residual cross-sectional reliance and slope heterogeneity, the Augmented Mean Group (AMG) econometric approach is employed.

**Results:**

Key outcomes from the mentioned estimation method unveiled that economic growth positively impacts obesity among all the study panels. Variably, unemployment was evidenced to have a palpable positive impact on obesity concerning Low-income economies whereas on the side of the Lower-middle income panel together with Upper-middle income economies and the aggregated panel, a significant negative relationship is observed with obesity. Further, urbanization enhanced obesity in the Low-income panel and the aggregated panel of African nations, whereas an adverse effect is identified in both the Lower-middle and Upper-middle economies in Africa. Moreover, except for Low-income African economies, all the other panels of African nations in terms of income levels were noted to have a significant negative effect on obesity from trade openness.

**Discussion:**

Finally, the long-run coefficients indicated that the OKC is valid among all panels of African countries. The study thus preferably suggests in African economies that addressing the inverted U-shape relationship between obesity and economic growth requires a multifaceted approach that considers the evolving dynamics of both factors. Policy makers should, therefore, aim to balance promoting economic growth and safeguarding public health through targeted interventions and long-term strategies.

## 1. Introduction

In the last two to three decades, obesity and overweight prevalence have increased globally. Since 1980, global obesity has more than doubled, and the majority of the world’s population now lives in nations where obesity kills more people than underweight (1–4). Obesity and overweight are risk factors for noncommunicable diseases (NCDs) such as cardiovascular diseases (CVDs), type 2 diabetes, musculoskeletal disorders, and cancers (5, 6). It is estimated that overweight people in 2016 accounted for more than 1.9 billion (39%) adults above the age of 18 around the world. Recent figures estimated that approximately 650 million of those who are overweight are obese according to the World Health Organization (4) fact sheet. Further, between 1975 and 2016, the global prevalence of obesity nearly tripled. Obesity has been identified to affect approximately 13% of the global adult population (4). In Africa, approximately 27% of adults aged 20 and above are overweight, with 8% being obese (6). While several African countries face the threat of communicable and poverty-related diseases (such as infant mortality, cholera, malnutrition, and malaria), the prevalence of chronic diseases continues to rise (7, 8). Obesity and overweight were originally only linked to high-income countries, but it is now common in low- and middle-income countries as well. Marshall (9) reported that developing countries are facing a “double burden” of disease. Thus, for instance, malaria and other infectious diseases affect people in several East African countries at an early age. Later in life, high-fat and processed food diets put them at risk of cardiovascular disease and diabetes (10). Evidently, according to some studies, the burden of chronic diseases in many developing nations will soon match the burden of acute infectious diseases (11, 12). The WHO predicts a doubling of mortality rates from ischemic heart disease in the African region by 2030 (13), as well as the largest increase in diabetes prevalence in developing countries by 2045 where economies are moving from low to middle income status (14).

The factors leading to the increase in obesity and overweight are multifaceted. Numerous studies have identified trade openness as one contributing factor to obesity and overweight (15, 16). There is a well-established conceptual framework that links trade openness to improved health outcomes, pointing to a rise in the availability of highly processed, energy-dense, nutrient-poor foods in developing nations as a result (17). Lin et al. (18) argued that the importation of processed food especially sugar is the reason for the increasing average body mass index (BMI) in countries. Empirical investigation by An et al. (19) revealed that, trade openness was found to be positively associated with obesity prevalence in countries, with the effect being strongest in emerging countries. Apart from trade openness, widely documented studies have identified unemployment or involuntary job loss as a factor that affects obesity prevalence among individuals (20–22). Others argue that long-term unemployment is correlated with increased risk of obesity as reported by Hofmeister (21). This finding is in tandem with the study of Antelo et al. (23), where authors found out that body mass index of respondents was impacted by long term unemployment or unemployed recently. Evidently, the prevalence of obesity in developed and developing countries, especially urban areas, has increased significantly in recent decades (24). Africa is not an exception to this recent urbanization which has led to rapid development in most of its regions. Despite the increasing prevalence rate of obesity, studies have projected that 20–25% of the African urban population are either obese or overweight (24, 25) and by 2025 it is anticipated that non-industrialized countries will account for three-quarters of the world’s obese population (26). Obesity and overweight have increased dramatically in recent years as a result of nutrition transition - dietary changes (fast food) and sedentary lifestyles. Rapid urbanization, adoption of western diets, changes in the lifestyle and intake of highly processed dietary foods contribute to increased obesity, lower physical activity and raised risk of metabolic and cardiovascular diseases. Apparently, low-and- middle income countries particularly in urban areas in Sub-Saharan African countries, are confronted with an expanding trend of obesity prevalence as reported by Biadgilign et al. (27).

Economic growth is an important element in the increase in the prevalence of obesity in most developed countries (28). With regards to economic growth, many studies conducted argued that obesity prevalence increases as individuals’ incomes increase to a certain threshold. The relationship is negative after that threshold point, resulting in an inverse U-shaped curve (28). Using Oman, Saudi Arabia, United Arab Emirates and Turkey as an example in a study conducted, a long-run coefficients results indicated that as personal income increases it would enable people to be able to afford more food which they might not need necessarily causing them to become overweight and obese. However, if they reach a certain threshold, they will want to eat healthier and reduce their caloric intake (29). Aggregate data from South Africa shows that there is an existence of obesity inequalities. That is, poor men are less likely to be obese than their richer counterparts (30). Nevertheless, individuals’ consumption reduces as they reach a specific financial level, combined with a better understanding of nutrition.

Although numerous empirical studies have been conducted to determine whether there is a causal relationship between economic growth, urbanization, trade openness, and unemployment, little attention has been paid to the nexus between the aforementioned factors that contribute to obesity in African countries. Concerning the studies conducted in Africa, Lartey et al. (31) employed multinomial and binomial logistics regressions to investigate the recent changes in the prevalence of obesity among older adults 50 years and above between 2007/08 and 2014/15 in Ghana. Peer et al. (32) in a study to determine the prevalence and determinants of overweight/obesity in the 25–74-year-old urban black population of Cape Town, and to investigate the changes between 1990 to 2008/09 used linear regression model analysis. Qualitatively, Agyemang et al. (33) examined the determinants of the epidemiology of obesity and overweight and the connection with diabetes and cardiovascular diseases in sub-Saharan African regions. Study findings revealed that South Africa was the most affected by obesity and overweight even though all African regions in the study experienced an increase. Socioeconomic level, gender, age, parity, physical inactivity, and increased caloric, fat, and sugar intake are all strong predictors of overweight and/or obesity, according to review findings of Steyn and Mchiza (5) in Sub – Saharan Africa. The use of multivariable logistic regression of pooled datasets from 27 countries (2003–2009) in sub-Saharan African countries established that maternal obesity is linked to a higher risk of neonatal death (34).

Despite the growing need to tackle obesity and overweight as well as other NCDs, studies conducted in Africa are limited compared to Western countries. This extant study compared to other related research therefore uniquely contributes to literature in three main folds.

- Firstly, this extant study takes into account a panel of African nations that have been subdivided into income levels. Specifically, only few studies concerning obesity have considered country specifics without taking into account the panel setting. For instance, Aydin (29) examined the effect of economic growth on obesity considering the most obese countries as distinct cross-sections. To the best of our familiarity, this is a novel study in Africa which examines the obesity Kuznet curve (OKC) framework considering trade, urbanization and unemployment as additional variables in both panel and sub-panel settings in terms of income levels. Both the aggregated panel and sub-panel classifications are considered in the study with the reason being that, conclusions as well as policy suggestions drawn from the outcomes concerning the aggregated panel, may not be true for all countries at different developmental stages as well as differences in economic structure, technical levels and resource endowment. Thus, employing the sub-panel classifications will enhance the study to determine whether factors unique to the countries within the sub-panels influence the affiliations between economic growth and obesity when examining the OKC hypothesis.
- Secondly, the Kuznet curve is a term that refers to Simon Kuznets’ (35) theory that, as a nation develops, a natural cycle takes shape in which quality initially rises and then falls. Nonetheless, as income increases and more resources are available to buy food, the Kuznet curve is extended in this study to form an obesity Kuznet curve. People eat more calories as a result, and obesity rates rise. However, as wealth continues to climb, people’s personal health becomes a more valuable possession and they start to lose weight, which raises their health levels.
- Thirdly, in terms of methods, the few related pieces of research which examined the liaison between economic growth and obesity both in and out of Africa ignored issues concerning residual cross-sectional reliance and heterogeneity among slopes which lead to spurious outcomes being provided. Thus, to resolve the mentioned issues which most related works have paid less attention, this recent study employs the Augmented Mean Group (AMG) estimation method to examine the OKC conjuncture concerning the relationship between economic growth and obesity.

The successive sections of this research work are thus organized as follows; Section 2 explores the various panel econometric methods used; Section 3 presents the empirical outcomes from the study’s analysis whereas Section 4 outlines the various discussions concerning the outcomes. Finally, the fifth segment concludes the study with policy suggestions or recommendations.

## 2. Methods

### 2.1. Data source and variable description

To achieve the objective of this extant research, panel time series data for African economies spanning from 1990 to 2020 has been utilized. The panel data employed consist of four main variables which includes obesity (measured in age-standardized prevalence of obesity among person aged 18+) as the response variable, economic growth (measured are gross domestic product (GDP), constant 2010 US dollars) as the main explanatory variables of interest whereas trade openness (measured as the value of imports plus exports as percentage of GDP) together with unemployment (measured as unemployment total percentage of labor force, modeled ILO estimate) and urbanization (measured as urban population as the share of total population) functions as control variables. Specifically, the employed panel data was extracted from World Health Organization’s Global Health Observatory database WHO (36) and World Bank Development Indicators WDI (37). Table 1 therefore summarily outlines the description of variables together with their respective data sources.

**Table 1:** Data source and description of variables.

Variable	Abbreviation	Measurement unit	Source
Obesity	obs	Age-standardized prevalence of obesity among person aged 18+	World Health Organization’s Global Health Observatory database (36)
Economic growth	gdp	Gross domestic product, constant 2010 US dollars	World Bank Development Indicators (37)
Trade openness	trd	Value of imports plus exports as percentage of GDP	World Bank Development Indicators (37)
Urbanization	urb	urban population as the share of total population	World Bank Development Indicators (37)
Unemployment	uemp	unemployment total percentage of labor force, modeled ILO estimate	World Bank Development Indicators (37)

Though the African continent is made up of 54 nations, taking into account the scarcity of data pertaining to some countries and certain variables, this extant study settled on 38 African countries. For instance, the countries which include Sao Tome and Principe together with Seychelles were opted out since they have insufficient data concerning economic growth. Further, Angola, Equatorial Guinea, Eritrea, Ethiopia, Lesotho and Malawi were also eliminated from the list of countries employed due to lack of sufficient data in the case of trade openness. Finally, the panel of 38 African countries selected for the study were subdivided into three sub-panels by using the gross national income (GNI) criterion from World Bank and Lending Group classification (38). Precisely, the income group classification includes, Low income (GNI less than 1,045 USD), Lower-middle income (GNI from 1,046 USD to 4,095 USD) and Upper-middle African countries (GNI from 4,096 to 12,695 USD). Numerically, the low-income African nations panel consisted of 18 countries, the Lower-middle income panel of African nations consisted of 15 countries whereas that of Upper-middle income African states’ panel comprised of five nations. Notably Table 1 outlines the details concerning the specific countries in each panel mentioned. Summarily, the list of countries employed for this extant study are outlined in Table 2.

**Table 2:** Classification of African countries by income levels.

Income level	List of selected countries	Number of countries
All countries	Algeria, Angola, Benin, Botswana, Burkina Faso, Burundi, Chad, Cote D’Ivoire, Republic of Congo, Democratic Republic of Congo, Equatorial Guinea, Eritrea, Eswatini (Swaziland), Ethiopia, Gabon, Gambia, Ghana, Guinea, Guinea-Bissau, Kenya, Lesotho, Madagascar, Malawi, Mali, Mauritania, Mozambique, Namibia, Niger, Nigeria, Rwanda, Senegal, Sierra Leone, South Africa, Togo, Uganda, United Republic of Tanzania, Zambia, Zimbabwe.	38
Low-income economies ($1,045 or less)	Burkina Faso, Burundi, Chad, Democratic Republic of Congo, Gambia, Guinea, Guinea-Bissau, Madagascar, Mali, Mozambique, Niger, Rwanda, Sierra Leone, Togo, Uganda, Eritrea, Ethiopia, Malawi.	18
Lower-middle income economies ($1,046 to $4,095)	Algeria, Benin, Republic of Congo, Cote D’Ivoire, Eswatini (Swaziland), Ghana, Kenya, Mauritania, Nigeria, Senegal, United Republic of Tanzania, Zambia, Zimbabwe, Angola, Lesotho.	15
Upper-middle-income economies ($4,096 to $12,695)	Botswana, Gabon, Namibia, South Africa, Equatorial Guinea.	5

### 2.2. Reasons for variables selection

Before estimating the panel econometric model as specified in Eq. (2), it is of much importance to discuss the essence of employing the study’s variables of interest in the midst of other variables which may deem potential. The study adopted three theories to discuss the reasons based on which the variables of importance were selected. The social-ecological model was adopted to explain the reason why economic growth and urbanization were selected for the study. The social-ecological model of health proposes that various factors affect an individual’s health, including biological and genetic factors, social connections and family relationships, environmental circumstances, and larger social and economic patterns (39). The association between economic growth and obesity is investigated in the literature via several transmission mechanisms, which is of interest to us. Economic growth, without a doubt, is the principal mechanism by which emerging economies may lift themselves out of poverty at the moment. Additionally, it has probably been one of the most significant influencers on health advancements throughout human history, as Egger et al. (40) reported. However, as a result of the law of diminishing returns, the benefits of sustained economic growth begin to dwindle and expenses begin to rise after a certain point (41). According to this argument, there may exist a theoretical GDP that is high enough to create adequate wealth, and promote good health, and happiness but not so high that it results in obesity and an unsustainable carbon footprint (42). Additionally, the impact of economic growth can be explained by technological advancements, which alter food prices and the economy’s structure. With the advancement of technology, a wider variety of foods has become more accessible. As a result, this might be viewed as a contributing factor to obesity, as it enables more convenient and excessive eating. On the other hand, as an individual’s financial situation improves, he or she is able to consume more food. Food consumption increases in this situation until income hits a turning point. As a result of this increase in food intake, obesity rates rise.

The social-ecological model further proposes that there are several levels of influence, and that these levels interact and reinforce one another. The model has been widely employed to elucidate how individuals’ physical activity and dietary choices are influenced by investigating the ever-changing environmental circumstances in which these behaviors take place (43). For instance, at the individual level factors such as genetics, behavior, and lifestyle choices can contribute to obesity. Urbanization can impact these factors by changing the food environment and opportunities for physical activity. The relationship between urbanization and obesity (also known as urban obesity), on the other hand, has recently been the subject of research, with recent findings suggesting that urbanization is responsible for shifts in dietary patterns and physical activity levels that tend to increase the risk for obesity (44). Urban obesity, particularly among the poor, is a problem all over Africa. For example, urban obesity is twice as high as it is in rural regions in Kenya, Senegal, and Ghana, and it is nearly three times as high across the continent—roughly a third of Africa’s urban population is obese. Africa confronts a growing obesity problem as a result of rapid urbanization and associated lifestyle changes. The proportion of Africans living in cities is expected to rise to 50% by 2030 and 60% by 2050 across the continent. As a result, increased urbanization is likely to be linked to lifestyle changes such as less physical activity. This is frequently accompanied by an increase in the consumption of high-calorie fast foods and sugary beverages. This combination has contributed to an increase in the prevalence of obesity in major areas and towns in developing countries, including Africa.

The political economy model can be used to explain the relationship between obesity and trade. This model suggests that trade policies and globalization can have significant impacts on the food environment and food systems, which in turn can contribute to the development of obesity (45, 46). Lin et al. (18) expressed in her study that an increase in trade flows would result in an increase in chronic illness and non-communicable diseases. The researcher further expressed that there are three nexus between trade and non-communicable diseases are highlighted in the literature: (a) growth of transnational food corporation (b) global food advertising and promotion (c) trade liberalization (47). Trade liberalization is the process of reducing tariffs and eliminating quotas. The emergence of a supermarket revolution and the quick global expansion of fast-food chains are examples of the growth of multinational food company connection, which is intertwined with broad transformation and control over the world’s food supply. The connection between worldwide food advertising and promotion highlights aggressive advertising and shifting food consumption habits. Furthermore, there is no doubt that higher trade openness results in increased economic growth, which may result in decreases in inequality and poverty rates, as well as a slew of other beneficial externalities. Nonetheless, in recent years, health experts have expressed worry that obesity and trade openness may be associated in some way as well. To be more specific, higher sugar consumption appears to be a significant method via which trade openness may have an impact on obesity rates. It has been argued that trade openness policies remove tariff barriers to imports of sugar-sweetened beverages, which may be connected to both obesity and related disorders (48). In addition, consumption of cheap, nutrient-poor foods is encouraged by the low cost and wide availability of imported goods with high fat and high sugar content (49). Thus, the passage of free trade agreements in Africa, such as the African Continental Free Trade Area (AfCFTA), may have further reduced the prices of refined sugar and other processed foods, which is compounded by allowing fast-food enterprises to expand into developing countries in regions such as Africa, negatively affecting individual consumption habits and leading to excessive food consumption. This may have influenced dietary changes that led to obesity.

Lastly, the social determinants of health model can be used to explain the relationship between obesity and unemployment. This model suggests that social and economic factors, such as income, education, and employment, can impact health outcomes, including obesity (50). When using the social determinants approach, it is important to focus on “the causes of the causes,” looking beyond the person to consider potential determinants that may arise at many levels. While it is obvious that social determinants include elements reflecting psychosocial (such as stress, occupational demands, and purely psychological constructs like depression and anxiety) and sociodemographic (such as gender, nativity, race/ethnicity, socioeconomic position) and characteristics, they also include more upstream elements like neighborhood traits, social structures, and the social environment. Unemployment can impact economic factors, such as income and access to resources, which can in turn impact food choices and physical activity levels. For example, individuals who are unemployed may have limited resources to purchase healthy foods or participate in physical activities that require a membership or equipment. According to a recent Gallup study, the longer someone is unemployed, the higher their chances of being obese, with rates as high as 32.7 percent after a year or more. Obesity is caused by unemployment, according to Cawley (51). He reinforced this argument by stating that unemployed individuals with lower salaries are more prone to purchase less expensive, but more fattening, items in order to save money. Furthermore, it was claimed that because obesity is a debilitating illness, obese employees are more likely to be less productive and, as a result, less likely to be hired, even when all other factors are included. If this results in decreased social position and income, it is believed that a vicious cycle will begin, with people consuming cheaper and fatter foods, which will, in turn, imperil their future employment opportunities. Given the foregoing rationales, economic growth, unemployment, trade openness, and urbanization are anticipated to have a significant link with obesity, and hence are appropriate variables to include in this study.

### 2.3. Model specification

Following the study of Grecu and Rotthoff (28) together with Aydin (29) this extant study based on the OKC framework seeks to estimate the non-linear (quadratic) relationship between obesity and economic growth. In order to reduce issues concerning the omission of variable bias (OMB), the study further incorporates potential variables of interest which include unemployment rate, and trade openness together with urbanization in a multivariate context. Thus, our proposed extended OKC model is specified as;

(1) $$obs=f\left(gdp,\;gdp^{2},\;\mathrm{uemp},\;trd,\;urb\right)$$

where obs together with gdp, ${gdp}^{2}\text{,}$ uemp, trd, and urb correspondingly represent obesity, economic growth, square of economic growth, trade openness and urbanization. In order to estimate the specified function in Eq. (1) econometrically, the explanatory variables are required to be expressed as a linear combination of their respective parameters. Moreover, due to potential issues of heteroscedasticity, data pertaining to each of the study variables are transformed into natural logarithm. Specifically, the log-transformation is executed prior to the estimation of the model so as to harmonize variance within the data employed. Log-transformation often helps to reduce the spread of data point of the explanatory variables in more constant variance across the range of the data. This harmonization of the variances addresses the issue of heteroscedasticity. In some cases, log-transforming variables in the model reduce the influence of extreme values or outliers in the data (52). Notably, heteroscedasticity is often caused by the presence of outliers and the log-transformation helps to mitigate their effects on the model. Based on these conditions augmented log-nonlinear OKC model in a panel setting is thus formulated as;

(2) $$\begin{array}{c} \mathrm{lnob}s_{\mathrm{it}}=\delta_{o}+\delta_{1}\mathrm{lngd}p_{i,t}+\delta_{2}\mathrm{lngd}{p^{2}}_{i,t}+\delta_{3}\mathrm{lnuem}p_{i,t}\\ +\;\delta_{4}\mathrm{lntr}d_{i,t}+\delta_{5}\ln urb_{i,t}+\varepsilon_{i,t} \end{array}$$

where $\delta_{o}$ is the constant term, $\delta_{1},\ldots \delta_{5}$ are the parameter estimates which capture the effect of economic growth and its square together with unemployment, trade openness and urbanization on obesity prevalence correspondingly. Specifically, for the OKC conjuncture to be valid or confirmed, the coefficient of GDP that is $\delta_{1}$ has to be positive whereas that of ${GDP}^{2}$ which is $\delta_{2}$ has to be negative. The positive $\delta_{1}$ together with the negative $\delta_{2}$ will therefore infer that, that as a country’s income rises and it undergoes economic development, the prevalence of obesity initially increases but eventually reaches a peak and starts to decline. This implies that obesity rates increase during the early stages of economic development, and then decrease as a country becomes more affluent. Thus, the level of income regarding countries with respect to specific panels in Africa will have attain in order to curb obesity is thus obtained using the relation;

(3) $$gdp=\exp\left(\frac{-\delta_{1}}{2\delta_{2}}\right)$$

### 2.4. Econometric estimation approaches

To estimate the study’s model as defined in Eq. (2), we first determined whether or not residual cross-sectional correlation and heterogeneity existed within the panel data used. Specifically, the presence or lack of residual cross-sectional connectedness and heterogeneity in a panel data environment has implications for the choice of additional econometric methodologies such as panel unit root and cointegration tests, as well as long-run estimate methods. Based on this claim, the Pesaran (53) cross-sectional reliance tests which include CD_P_ test, CD_LM_ and the CD_LM-ADJ_ tests were used. Concerning the heterogeneity test, the Pesaran and Yamagata (54) homogeneity test was also used in the investigation. Bearing in mind the possibility of residual cross-sectional reliance and heterogeneity in a panel data setting, the study went on to look at the integration properties of the used variables using the cross-sectional Augmented Dickey Fuller (CADF) and the cross-sectional Im, Pesaran, and Shin (CIPS) by Pesaran (55). Apart from looking into the stationarity characteristics, we also looked into the long-run correlations between the research variables using the Westerlund and Edgerton (56) bootstrap cointegration test and the Durbin–Hausman panel cointegration (57). The study calculated the long-run relationship between a series of variables using the Augmented Mean Group (AMG) methodology developed by Bond and Eberhardt (58) once the conditions relevant to the afore-mentioned preparatory procedures were satisfied.

Based on shreds of evidence, the AMG estimation approach takes heterogeneity across countries into account and also enables the parameters of non-stationary variables to be examined. Theoretically, the AMG long-run estimation procedure is based on two main stages. From the first stage of the AMG method, the study’s proposed model as specified in Eq. (2) is estimated using T-1 dummies in a differenced form (first difference) through the following panel regression model specified as;

(4) $$\begin{array}{c} \ln\Delta obs_{\mathrm{it}}=\delta_{i}+\delta_{1}\ln\Delta gdp_{\mathrm{it}}+\delta_{2}\ln\Delta gd{p_{\mathrm{it}}}^{2}+\delta_{3}\ln\mathrm{\Delta uem}p_{\mathrm{it}}\\ +\;\delta_{4}\ln\Delta trd_{\mathrm{it}}+\delta_{5}\ln\Delta urb_{\mathrm{it}}+\overset{T}{\underset{t=1}{\sum }}\emptyset_{t}\left(\Delta D_{t}\right)+\varepsilon_{\mathrm{it}} \end{array}$$

where $\Delta D_{t}$ denotes the first difference order with T-1 time dummies, $\Theta_{t}$ represents the coefficients of $\Delta D_{t}$ , $\delta_{i}$ is the constant term whereas $\delta_{1}$ ,…, $\delta_{5}$ capture the parameter estimates of the differenced study variables.

With the second stage, the parameter $\emptyset_{t}$ is transformed to η_t (i.e., $\emptyset_{t}$ = $\eta_{t}$ ) then express in a common dynamic process as follows;

(5) $$\begin{array}{c} \ln\Delta OBS_{\mathrm{it}}=\delta_{i}+\delta_{1}\ln\Delta gdp_{\mathrm{it}}+\delta_{2}\ln\Delta gd{p_{\mathrm{it}}}^{2}+\delta_{3}\ln\mathrm{\Delta uem}p_{\mathrm{it}}\\ +\;\delta_{4}\ln\Delta trd_{\mathrm{it}}+\delta_{5}\ln\Delta urb_{\mathrm{it}}+\eta_{t}\left(d_{t}\right)+\varepsilon_{\mathrm{it}} \end{array}$$

$\begin{array}{l} \mathrm{ln\Delta OB}S_{\mathrm{it}}-\eta_{t}\left(d_{t}\right)=\delta_{i}+\delta_{1}\ln\Delta {gdp}_{\mathrm{it}}\\ +\delta_{2}\ln\Delta {{gdp}_{\mathrm{it}}}^{2}+\;\delta_{3}\ln\Delta {\mathrm{uemp}}_{\mathrm{it}}+\;\delta_{4}\ln\Delta {trd}_{\mathrm{it}}+\delta_{5}\ln\Delta {urb}_{\mathrm{it}}+\varepsilon_{\mathrm{it}}\text{,} \end{array}$ and *d*^t^ is represents the standard dynamic process and estimated coefficients of each dummy.

We can, therefore, deduce that, the cross-section panel model with η_t as specified in Eq. (5) is adjusted before estimating the average parameter estimates in terms of country specifics. Thus for each explanatory variable specified in the study’s model, the parameters to estimate can be computed using the relation:

(6) $${{\hat{\delta }}_{i}}_{AMG}=\frac{I}{N}\overset{N}{\underset{i=1}{\sum }}{\hat{\delta }}_{i}$$

where ${{\hat{\delta }}_{i}}_{AMG}$ denotes the AMG estimator.

Specifically, the AMG assumes that the underlying relationship between the response variable and explanatory variables has the same slope across all individual panels. Further the AMG model assumes that there are no unobserved individual-specific fixed effects that are correlated with the explanatory variables. This assumption allows for pooling the data across individuals to estimate the common slope coefficients. Moreover, the error term in the AMG model is assumed to be uncorrelated across time and individuals. This assumption thus implies that there is no serial correlation or autocorrelation in the error term which ensures the efficiency of the estimation. Furthermore, the error terms are assumed to be uncorrelated across individual cross-sections. This thus implies that there is no cross-sectional correlation or contemporaneous correlation among the error terms of different cross-sections.

### 2.5. Descriptive statistics

Summarily, Table 3 outlines the descriptive statistics pertaining to the study variables (obesity, economic growth, trade openness, unemployment and urbanization) based on their respective means, standard deviations, skewness and kurtosis together with Jarque-Bera test of normality. Specifically, a summary of the descriptive statistics as outlined in Table 3 is performed based on the panel of African economies and three sub-panels in terms of income levels. Due to the larger nature of values pertaining to the mean and standard deviation values of economic growth and urbanization we converted them into millions (10^6). Considering the outcomes from the mentioned table, it is evident that the variable GDP (economic growth) has the highest mean estimates across all panels of African economies followed urbanization and then trade openness with unemployment having the least average value from one country group to the other. According to Mensah et al. (59), the implication of GDP having the highest mean values compared to the other variables may be due to the aggregate demand for high incomes, tax exemptions, devaluations, minimal government expenditure and ultimately low-interest rates evidenced in most of the African economies regardless of their respective income levels. On the side of the increase in production, this outcome in terms GDP having the highest mean value could be due to the surge in comprehensive supply in terms of increased investment. Generally, it is evident there exist some variabilities pertaining to the results from the sub-country panels. Specifically, except for obesity and urbanization, upper-middle-income African states seem in relation of the other groups of Africans comparatively seems to have the highest level of economic growth, trade and unemployment. An implication to this is that, as African economies conduit from low-income level to upper-income level states, the degree of economic growth together with trade and unemployment progresses. Nonetheless, obesity being the main variable of interest seems to be averagely high in Upper middle-income African economies followed by Low-income panel of African nations and then Lower middle African states though the difference existing between the figures reported are very marginal. This outcome descriptively supports the study of Templin et al. (60) who stated that, in most low and upper-middle-income countries, the prevalence of obesity is higher among wealthier individuals than among the poorer.

**Table 3:** Summary of descriptive statistics.

Panel	Variable	Mean	Standard Dev.	Skewness	Kurtosis	JB-test
Aggregated panel						
	obs	29.320	2.683	−0.124	2.545	11.705^***^
	gdp	27682.262	66198.802	4.016	19.783	15059.590^***^
	trd	64.861	27.627	0.975	3.634	183.012^***^
	uemp	8.031	7.409	1.367	3.908	360.784^***^
	urb	5.356	9.555	4.497	28.501	31810.41^***^
L-I panel						
	obs	29.577	2.716	−0.781	2.894	44.450^***^
	gdp	6578.084	6331.396	1.961	7.298	613.740^***^
	trd	54.482	21.475	1.261	5.128	197.507^***^
	uemp	3.485	2.314	1.300	4.448	160.724^***^
	urb	3.722	5.284	3.510	16.384	4140.780^***^
L-MI panel						
	obs	28.923	2.598	0.512	2.968	20.322^***^
	gdp	35119.606	66895.854	3.593	2.071	5185.011^***^
	trd	66.683	28.547	1.213	4.290	146.093^***^
	uemp	8.880	6.928	1.303	3.818	144.411^***^
	urb	8.234	12.758	3.412	16.480	4414.045^***^
UP-MI panel						
	obs	29.817	2.689	1.190	3.428	63.539^***^
	gdp	67195.297	118894.708	1.763	4.539	89.495^***^
	trd	90.171	23.169	2.151	6.564	873.700^***^
	uemp	18.913	6.554	1.428	4.001	99.171^***^
	urb	1.0501	0.589	1.141	3.875	36.103^***^

L-I together with L-MI and U-MI also means Low income., Lower-Middle income and Upper-Middle income. Further, the JB-test denotes Jarque-Bera test. Due to the large values pertaining to the means and standard deviations of GDP and URB, they were correspondingly converted into millions (10^6^).

*** Represents 1% level of significance and leads to the rejection of the null hypothesis of normality.

In addition, for the data pertaining to the study variables to be normally distributed, the Jarque-Bera (JB) test together with the skewness and kurtosis examinations are executed. Notably, data concerning a specific variable is said to be normally distributed if the skewness together with the kurtosis are, respectively, 0 and 3 approximately Westfall (61) whereas the JB test is expected to be substantially significant. Summarily, it is evident from Table 2, that none of the variables employed seems to follow the normal distribution since their respective Kurtosis and Skewness values do not meet the thresholds 0 and 3 correspondingly. This is as well supported by the JB test which leads to the rejection of the null conjuncture that data pertaining to the employed variables follows the normal distribution. Since the data for the corresponding variables utilized in the study do not follow the normal curve, the study employs non-parametric approaches to analyze the employed data.

Moreover, the employment of multiple explanatory variables in our proposed model as specified in Eq. (2) can give rise to issues pertaining to high interdependencies (multicollinearity). Thus, in order to verify as to whether there exist any issues of multi-collinearity among the explanatory variables a correlation matrix together with variance inflation factor (VIF) and tolerance tests are executed. Considering the outcomes from the correlation matrix, there exists a weak correlation among each pair of explanatory variables across all the employed panels. This thus gives the indication that the corresponding variables are not highly dependent on each hence there exists no issue of multicollinearity. Further, the respective values of the VIF and Tolerance with respect to the explanatory variables are correspondingly less than the threshold of 5 and as well greater than 0.2. This outcome as a result supports the verdict from the correlation matrix that there exist no multicollinearity issues. The outcomes concerning the correlation matrix together with the VIF and tolerance tests are highlighted in Table 4.

**Table 4:** Multicollinearity examination.

Aggregated panel						
	lngdp	lnuemp	lnurb	lntrd	VIF	1/VIF
lngdp	1.0000				3.320	0.302
lnuemp	0.215	1.000			3.270	0.306
lnurb	0.406	−0.179	1.000		1.810	0.554
lntrd	−0.006	0.499	−0.207	1.000	1.360	0.735
L-I panel						
lngdp	1.000				2.785	0.359
lnuemp	−0.073	1.000			1.320	0.756
Inurb	0.184	0.152	1.000		3.982	0.251
lntrd	0.074	0.269	0.189	1.000	1.121	0.892
L-MI panel						
lngdp	1.000				2.151	0.465
lnuemp	−0.178	1.000			1.730	0.578
Inurb	0.352	−0.336	1.000		1.565	0.640
lntrd	−0.217	0.101	−0.309	1.000	1.380	0.724
U-MI panel						
lngdp	1.000				2.600	0.385
lnuemp	0.128	1.000			2.510	0.224
lnurb	0.425	0.269	1.000		3.6600	0.273
lntrd	−0.336	−0.130	−0.498	1.000	4.460	0.224

### 2.6. Cross-sectional spatial pattern of obesity in Africa (1990–2020)

**Figure 1:**
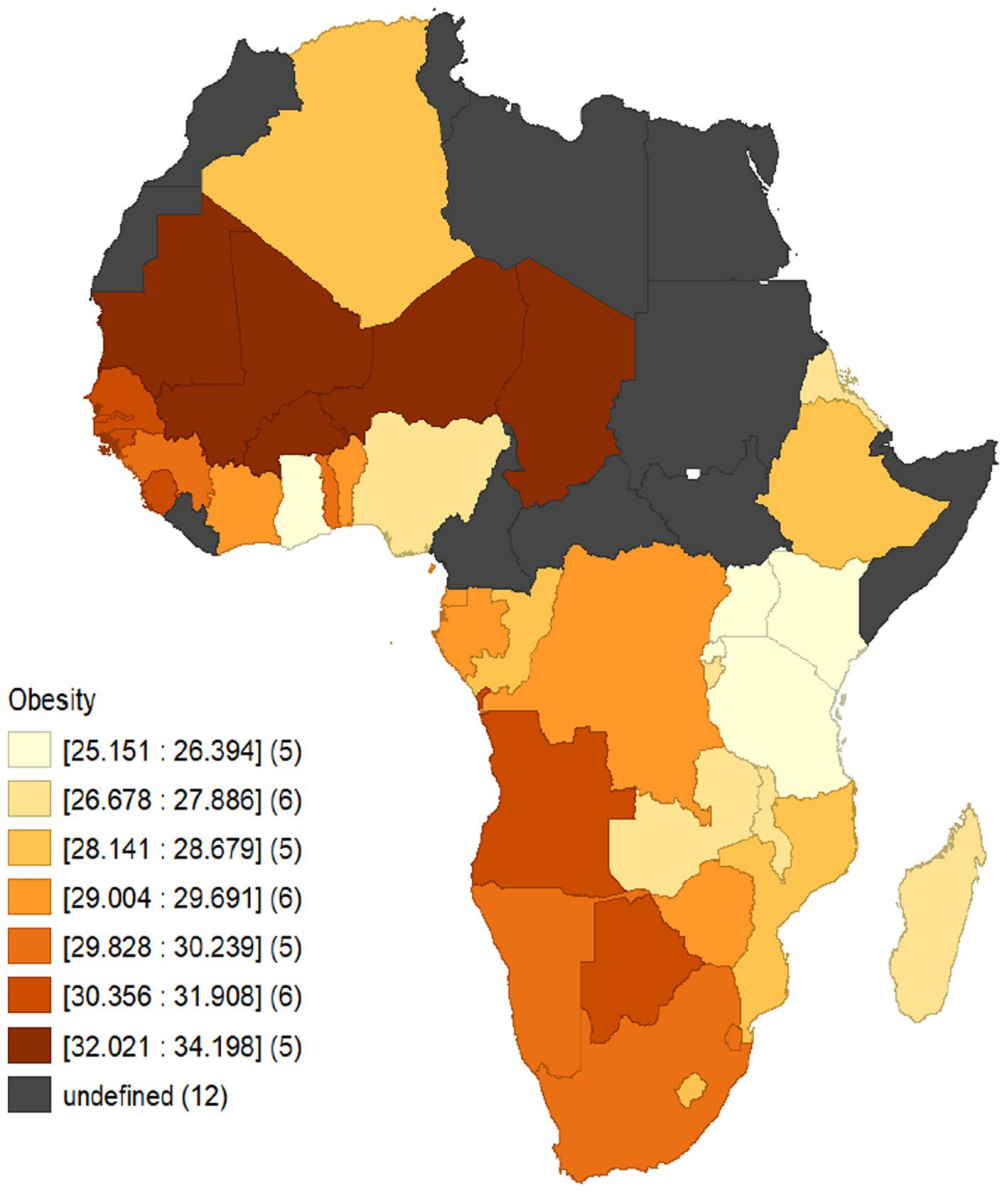
Spatial distributional pattern of obesity across African economies from 1990 to 2020.

With the aim examining the liaison between obesity and economic growth considering the case of African economies, the distributional pattern of obesity is analyzed from a spatial perspective considering a data span from 1990 to 2020. Figure 1 thus illustrates the spatial pattern of obesity across the African countries employed in the study. It is evident that among the panel of 38 African economies employed, Mauritania together with Burkina Faso, Mali, Niger and Chad averagely recorded the highest level of obesity within the range 32.021–34.198. This was followed by Senegal, The Gambia, Guinea Bissau, Sierra Leone, Botswana and Angola who as well recorded average level of obesity from 30.356–31.908. Further, countries which includes, Guinea, Togo, Namibia, South Africa, Eswatini, Cote D’Ivoire, Benin, Equatorial Guinea, Gabon, D.R.Congo, Zimbabwe, Lesotho, Algeria, Congo, Mozambique, Ethiopia, Nigeria, Zambia, Malawi, Eritrea, Madagascar, and Burundi averagely fell within the obesity prevalence range of 26.678–30.239. Finally, Ghana together with Uganda, Kenya, Tanzania and Rwanda comparatively recorded the least level of obesity prevalence with a recorded range of 25.151–26.394. Summarily, it evident that from the spatial pattern that, obesity prevalence differs or vary across the African economies employed in the study. These variations may be attributed to dietary patterns, lifestyle choices, access to healthcare and socioeconomic conditions. As well the countries involved are characterized by different income levels, they experience economic growth and urbanization. As a result, there may be an increase in obesity rates due to changes in dietary habits, reduced physical activity and increased availability pf processed foods. Nonetheless, this trend may not be uniform across all countries since cultural and regional variables together with developmental stages also play vital roles.

## 3. Results

### 3.1. Cross-sectional reliance and homogeneity tests

Prior to estimating the proposed model, the study first examined whether there exist any issues concerning residual cross-sectional reliance together with heterogeneity. The results pertaining to both the cross-sectional dependency and heterogeneity tests are outlined in Table 5. Phillips and Sul (56) have reported that cross-sectional reliance among cross-sections in a panel data setting becomes substantial, the efficiency of the estimated outcomes can be greatly reduced. Thus, taking into account the residual cross-sectional dependency test, the CD_P_ test together with CD_LM_ and CD_LM-ADJ_ tests have been employed. Outcomes from the mentioned CD tests depict that, the null conjuncture of cross-sectional independence is rejected among all the variables across the country panels. This, therefore, gives an indication the data containing the variables do not suffer from any issues of residual cross-sectional connectedness. Further, the Pesaran and Yamagata test of homogeneity is employed to examine whether there exist any issues concerning slope homogeneity or not. Relying on the outcomes from the delta Δ~ and adjusted delta tilde Δ~ -adj of the homogeneity test, the null hypothesis of slope homogeneity is rejected for all the series of variables among the country panels of African countries employed in the study. This, therefore, gives the indication that there exist issues pertaining to slope heterogeneity among the variables employed across all the country panels in terms of income levels; this leads to the employment of second-generation panel econometric approaches which are robust to the evidenced issues concerning residual cross-sectional reliance and slope heterogeneity.

**Table 5:** Residual cross-sectional reliance and slope homogeneity tests.

Variable	Cross-sectional dependence tests			Homogeneity test	
	CD_P_ test	CD_LM_ test	CD_LMadj_ test	Δ~	Δ~ -_adj_ test
L-I panel					
Inobs	12.207^***^	25.591^***^	25.232^***^	24.490^***^	27.500^***^
Ingdp	48.043^***^	17.463^***^	10.269^***^	20.281^***^	22.773^***^
Inuemp	24.394^***^	46.936^***^	63.511^***^	21.632^***^	24.290^***^
Inurb	54.382^***^	15.522^***^	6.771^***^	27.548^***^	30.933^***^
Intrd	12.822^***^	32.181^***^	36.766^***^	10.684^***^	11.996^***^
L-MI panel					
Inobs	19.654^***^	33.534^***^	34.293^***^	22.887^***^	25.700^***^
Ingdp	47.605^***^	30.472^***^	28.485^***^	23.267^***^	26.126^***^
Inuemp	14.403^***^	314.1^***^	30.020^***^	22.637^***^	25.418^***^
Inurb	56.911^***^	33.500^***^	33.994^***^	23.996^***^	26.945^***^
Intrd	16.856^***^	26.264^***^	21.228^***^	14.794^***^	16.612^***^
UP-MI panel					
Inobs	16.239^***^	18.440^***^	13.394^***^	15.508^***^	17.414^***^
Ingdp	16.285^***^	29.474^***^	10.666^***^	10.524^***^	11.818^***^
Inuemp	10.273^***^	16.628^***^	13.257^***^	7.503^**^	8.425^**^
Inurb	16.161^***^	26.781^***^	9.241^***^	12.703^***^	14.264^***^
Intrd	11.103^***^	33.450^***^	13.141^***^	14.513^***^	12.822^***^
Aggregated panel					
Inobs	7.975^**^	16.211^***^	68.314^***^	46.831^***^	52.586^***^
Ingdp	15.185^***^	13.295^***^	45.698^***^	36.770 ^***^	41.289^***^
Inuemp	13.395^***^	19.300^***^	89.942^***^	33.625^***^	37.757^***^
Inurb	31.387^***^	12.725^***^	42.060^***^	36.929^***^	41.468^***^
Intrd	10.671^***^	17.833^***^	79.001^***^	19.695^***^	22.115^***^

*** Represents 1% level of significance whereas.

** Denotes significance level at 5%.

### 3.2. Panel unit root tests

With the presence of issues concerning cross-sectional residuals and slope heterogeneity, the study in this section investigated the integration properties of the series of variables using the CIPS and CADF panel unit root tests. Outcomes concerning the mentioned unit root tests are therefore outlined in Table 6. Results from the aforesaid table depict that, all the variables among the various income level country groupings are characterized by homogeneous order of integration. This indicates that all variables employed in this extant study are non-stationary at levels but becomes stationary when differenced. We thus accomplish that, in the presence of cross-sectional residual correlations and slope heterogeneity issues, all series of variables have the same order integration [I(1)]. The outlined outcome pertaining to the employed panel unit root examinations leads to the test of cointegration among variables which will be unveiled in the succeeding section.

**Table 6:** Results from CADF and CIPS panel unit root tests.

Variable	CADF test		CIPS test		Decision
	Levels	Δ	Levels	Δ	
	(Const. and Trend)	(Const. and Trend)	(Const. and Trend)	(Const. and Trend)	
L-I panel					
Inobs	−2.078	−3.672^***^	−2.267	−5.190^***^	I(1)
Ingdp	−2.527	−3.803^***^	−2.513	−4.631^***^	I(1)
Inuemp	−1.600	−3.038^***^	−1.600	−3.038^***^	I(1)
Inurb	0.214	−3.304^***^	−1.038	−3.304^***^	I(1)
Intrd	−2.710	−3.608^***^	−3.561	−5.667^***^	I(1)
L-MI panel					
Inobs	−1.752	−3.233^***^	−1.750	−5.312^***^	I(1)
Ingdp	−1.833	−2.894^***^	−1.749	−4.209^***^	I(1)
Inuemp	−1.274	−3.046^***^	−1.274	−3.046^***^	I(1)
Inurb	−0.603	−2.960^***^	−0.603	−2.960^***^	I(1)
Intrd	−2.425	−3.852^***^	−2.263	−5.062^***^	I(1)
UP-MI panel					
Inobs	−0.838	−4.177^***^	−0.838	−4.177^***^	I(1)
Ingdp	−1.940	−4.550^***^	−1.940	−4.550^***^	I(1)
Inuemp	−2.098	−3.340^***^	−2.098	−3.340^***^	I(1)
Inurb	−1.693	−2.926^*^	−1.693	−4.050^***^	I(1)
Intrd	−1.982	−3.190^***^	−1.445	−5.143^***^	I(1)
Aggregated panel					
Inobs	−1.606	−2.693^***^	−1.935	−4.674^***^	I(1)
Ingdp	−2.327	−3.328^***^	−2.113	−4.499^***^	I(1)
Inuemp	−1.435	−3.036^***^	−1.435	−3.036^***^	I(1)
Inurb	−0.064	−2.963^***^	−0.064	−2.963^***^	I(1)
Intrd	−2.551	−2.799^***^	−1.799	−2.881^***^	I(1)

* Represent significance level at 10%.

*** Represent significance level at 1%.

### 3.3. Panel cointegration outcomes

Bearing in mind variables being integrated of the same order, we further examined whether there exists any long-run relationship (cointegration) among the response and explanatory variables specified in the study’s proposed panel model. Thus, the Westerlund-Edgerton panel cointegration approaches are utilized. Table 7 thus outlines the outcomes pertaining the mentioned methods of panel cointegration. Outcome from the mentioned test of cointegration exhibits that the null hypothesis of non-cointegration among the series of variables is rejected across all the country groupings in terms of income levels. The rejection of the null conjuncture of non-cointegration is due to the fact that all the group and panel statistics with regards to the Westerlund-Edgerton cointegration technique are statistically significant. This thus gives the indication that there exists a long-run relation (cointegration) among the employed variables which thus needs to be estimated.

**Table 7:** Results from Westerlund-Edgerton bootstrap panel cointegration test.

Panel	Group test statistics				Panel test statistics			
	$G_{\tau }$		$G_{\alpha }$		$P_{\tau }$		$P_{\alpha }$	
	*Z*-test value	Robust *p*-value	*Z*-test value	Robust *p*-value	*Z*-test value	Robust *p*-value	*Z*-test value	Robust *p*-value
L-I panel	−2.197^***^	0.005	−1.821^***^	0.002	−5.804^****^	0.000	−1.071^***^	0.001
L-MI panel	−3.171^***^	0.000	−6.714^***^	0.006	−11.720^***^	0.000	−5.416^****^	0.001
U-MI panel	−2.728^***^	0.009	−4.635^***^	0.021	−12.403^***^	0.000	−3.119^***^	0.025
Aggregated panel	−3.090^***^	0.000	−9.293^***^	0.002	−13.408^***^	0.000	−6.227^***^	0.004

*** Denotes significance level at 1%.

### 3.4. Panel long-run estimation analysis

In the case where the series of variables are integrated at the same order and as well cointegrated, a vital corollary is to further estimate the evidenced long-run liaison amid the study variables. Thus, considering the existence of issues concerning residual cross-sectional residuals together with slope heterogeneity (as witnessed in Table 5), the Augmented Mean Group (AMG) estimation technique is employed. The outcomes from the AMG approach for all the study panels is as a result outlined in Table 8. By comparing the outcomes from the various panels including the aggregated panel (L-I, L-MI, and UP-MI), the effect of economic growth on obesity is significantly positive across all the panels though the weight of the impact is very substantial in Lower middle-income African economies followed by Upper-middle income countries in Africa. In the case of unemployment, a palpable positive impact is evidenced on obesity in the L-I income panel whereas on the side of L-MI together with UP-MI and the aggregated panel a significant negative relation is observed with obesity. Furthermore, in L-I panel and the aggregated panel of African nations, a positive significant effect on obesity from urbanization is established; nonetheless, the former variable (urbanization) is evidenced to have a negative influence on the latter (obesity) in both the L-MI and UP-MI economies in Africa.

**Table 8:** AMG long -run estimation.

Response var. lnobs			
Variable	Coefficient	*p*-value	OKC hypothesis
Low-income economies			
Lngdp	0.112	0.000	Supported
Lngdp2	−0.265	0.000	
Lnuemp	0.062	0.040	
Lnurb	0.173	0.000	
lntrd	0.204	0.000	
Turning point (OKC)	1.235		
Wald Chi-square	30.570	0.000	
RMSE (sigma)	0.002		
Lower-middle income			
Lngdp	0.402	0.000	Supported
Lngdp2	−0.051	0.031	
Lnuemp	−0.227	0.000	
Lnurb	−0.323	0.000	
lntrd	−0.131	0.001	
Turning point (OKC)	51.479		
Wald Chi-square	16.620	0.005	
RMSE (sigma)	0.002		
Upper-middle income			
Lngdp	0.201	0.000	Supported
Lngdp2	−0.235	0.000	
Lnuemp	−0.072	0.002	
Lnurb	−0.093	0.040	
lntrd	−0.370	0.000	
Turning point (OKC)	1.534		
Wald Chi-square	116.690	0.0000	
RMSE (sigma)	0.003		
All countries			

Lngdp	0.978	0.000	Supported
Lngdp2	−0.151	0.007	
Lnuemp	−0.095	0.015	
Lnurb	0.144	0.023	
lntrd	−0.139	0.413	
Turning point (OKC)	25.241		
Wald Chi-square	29.240	0.002	
RMSE (sigma)	0.003		

**Figure 2:**
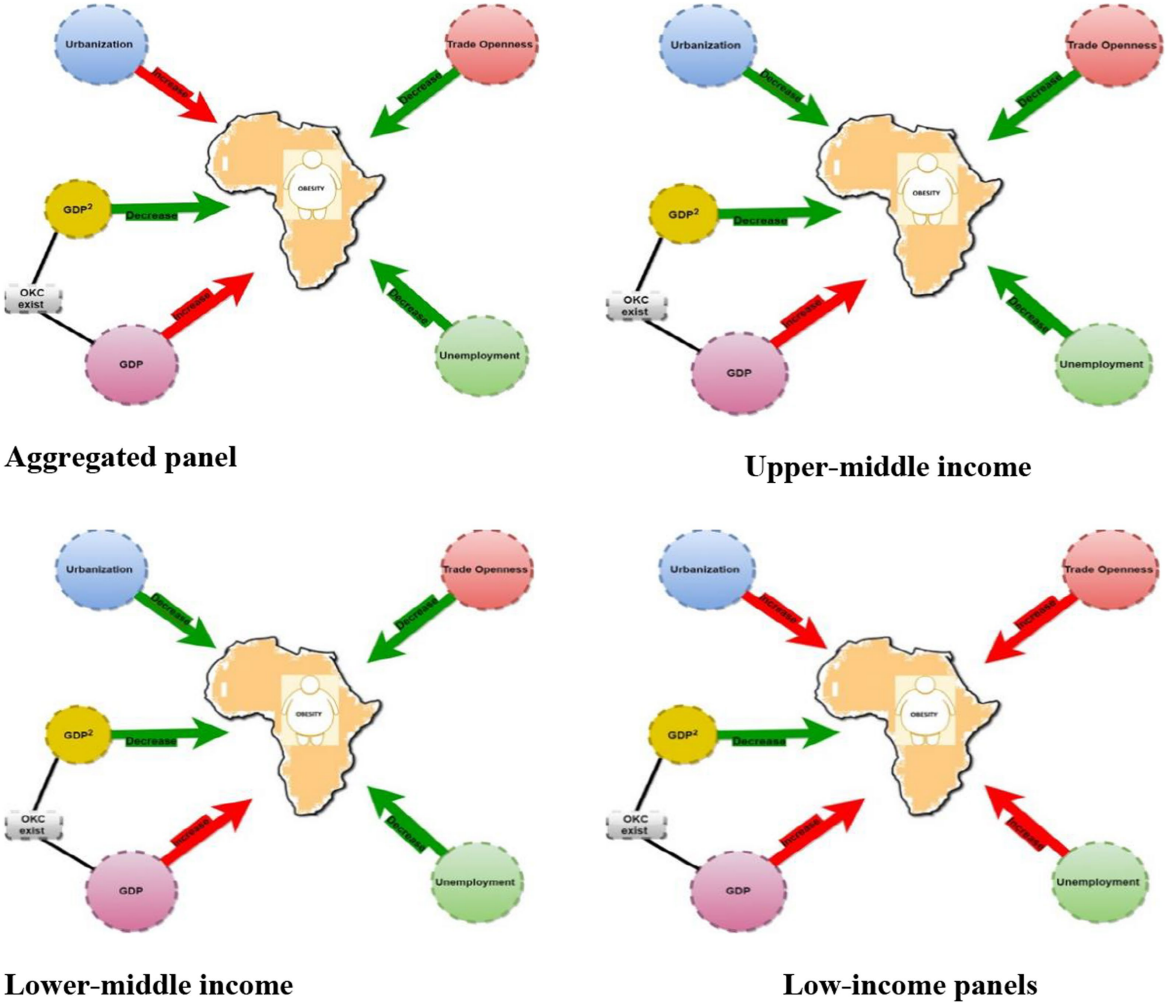
Path diagram illustrating the existence of OKC and effects of GDP, unemployment, trade openness and urbanization on Obesity based on income level distribution in Africa. NB: the green arrow indicates a specific variable leads to the decline in obesity whilst a red arrow as well shows a specific variable enhances or surges the prevalence of obesity.

Finally, considering the case of trade openness, with the exception of the L-I economies, all the other country groups (L-MI, UP-MI and aggregated panel of African nations) are characterized by noteworthy negative effects from openness in trade to obesity. Interestingly, the long-run liaison coefficients confirm that the OKC is valid among all the country panels employed in Africa. The implication concerning the validity of the OKC conjuncture among all the panels is that economic growth in African countries regardless of income levels surges the personal income of individuals causing them to be able to afford more food (thus increasing their calories which leads to weight). Nonetheless, after reaching a turning point of income, citizens in the various countries in Africa will have the desire to be healthier, decreasing their caloric intake (and thus reducing their weight as they increase their health levels). Considering the existence of the OKC conjuncture among the panels of African economies, the threshold points for the various panels were as well computed using the parameters estimates of economic growth and its square. Evidently, the threshold point for the aggregated panel of African nations was obtained at 25.241 whereas in the case of the sub-panels in terms of income levels, the threshold points of 1.235, 51.479, and 1.534 were linked to low income, lower-middle income and Upper middle income African nations correspondingly. Aside from estimating the long-run effect of economic growth, urbanization, unemployment and trade openness on obesity, the AMG estimation outcome as outlined in Table 8 comes with post-estimation results based on the Root Mean Square (RMSE) and the Wald Chi-square tests. Results pertaining to the mentioned post-estimation methods exhibit a good sign of fitness for the proposed obesity model among all the panels employed in the study. The summary of the long-run estimation concerning the effect of economic growth, urbanization, unemployment and trade openness together with the confirmation of the OKC conjuncture among the study panels are as well illustrated in Figure 2.

## 4. Discussion

Some findings from prior research were verified in this study, although not all. The long-run estimation outcomes per income level show that there is a clear pattern of obesity Kuznets curve which is valid for the aggregated panel, upper-middle-income countries, lower middle-income countries and low-income countries. This study result implies that an increase in income causes people to consume or receive more nutrients than they require which is as a result of economic growth in the country. As individuals consume more and more food because they are able to afford it, their caloric intake increases which result in an increase in their weight. However, if they reach a certain level of income, people will want to eat healthier and reduce their caloric intake (and thus decrease their weight as they increase their health levels). The study results present clear evidence supporting obesity Kuznets curve relationship between economic growth and obesity simply means that as countries’ income increases, the obesity prevalence, and overweight rates increase; thus, health risk increases as there is an increase in incomes. This is consistent with studies conducted by (28, 29, 62, 63) which showed there was an existence of OKC in countries with increased GDP. Obesity rises in tandem with rising affluence, according to our findings. When people initially get out of poverty, their better incomes allow them to increase their calorie consumption by eating more food or eating more densely. It’s also likely that as their wages increases, they’ll exercise less, drive instead of walking, or work in a sedentary job instead of being active all day. However, as earnings increase above a certain threshold, people’s ability to purchase and consume healthier foods, as well as their ability to increase physical activity, improves, lowering obesity rates. Mathieu-Bolh and Wendner (64) argued that weight consciousness reinforces the obesity Kuznets curve, thus, as a country develops, the income effect enhances average food spending and calorie intake. The dynamic status effect, on the other hand, makes low-calorie food more appealing than high-calorie food, which tends to reduce calorie consumption. Individuals will switch low-calorie items for high-calorie foods if the dynamic status effect outweighs the income effect, resulting in lower calorie consumption and lower average obesity rates. In contrast with this study is a study by Talukdar et al. (65) who discovered a positive association between population obesity prevalence and national income, but no indication that the relationship, while diminishing, truly turns negative at higher income levels (obesity Kuznets curve). Further, the OKC conjuncture suggest as countries income increases, the prevalence of obesity initially rises but eventually starts to decline. Relying on this concept, the various threshold points with respect to the panel of African countries employed were analyzed. The threshold points for the sub-panels which includes the low income, lower-middle income and upper-middle income African economies were 1.235, 51.479, and 1.534 correspondingly whereas for the aggregated panel of African countries a threshold point of 25.241 was evidenced. The outlined threshold points imply that at income levels of 1.235, 51.479, and 1.534 (all measured in GDP *per capita*), the prevalence of obesity in low income, lower-middle income and upper-middle income African nations begins to decline, whereas an income level of 25.241 (measured in GDP *per capita*) is expected for the relationship between income and obesity to transition from positive to negative in the aggregated panel of African economies. Comparatively, among the sub-panels, Lower-middle income African nations had the highest threshold point whereas African countries with low and upper-middle income levels were characterized by the least threshold points. This analysis demonstrates that, countries within the Lower-middle income African panel will require more years to reach their threshold point compared to the African countries within the Low and Upper-middle sub-panels.

Unemployment from the long-run estimation is witnessed to be positively correlated with obesity in low-income countries but negative in other panels. This result demonstrates that in the case of low-income countries perhaps unemployed people consume more caloric or energy-dense food and stay indoors without working out or engaging in any form of physical activity that requires the use of more energy. This, therefore, increase health risk which is a result of overweight or obesity. Individuals trade off the inconvenience of being overweight or obese for the pleasure of eating and leading a sedentary lifestyle. Furthermore, unemployment has been associated with depression in some studies as reported by Latif (66) and it has been evidenced that it has the tendency of promoting weight gain. Also, unemployed persons are more likely to lose money. As a result, their consumption of a high-cost, high-nutrient diet may decrease, resulting in weight gain which is in line with studies conducted by Latif (66). On the other hand, in the case of the aggregated panel, upper middle income and lower - middle income it was observed that unemployment had a negative relationship with obesity. This can be attributed to the fact that unemployed persons have more time to engage in healthful activities such as physical activity. This physical activity could help them lose weight by increasing their expenditure on calories. During unemployment, an individual’s financial resources (for example, from resource pooling within a spouse or family) may alleviate budgetary constraints on food quality.

The study results further revealed that urbanization was positively associated with obesity in the aggregate panel and low-income countries whiles negatively correlated in upper-middle-income and low-middle-income countries. The positive relationship can be attributed to the fact that rapid urbanization in most countries (low income) may have contributed to the swift change in lifestyle and way of life of the people. For instance, people prefer fast foods which are readily available and prefer sedentary jobs which they believe are less demanding and reputable. In addition, as most countries develop western tastes are much preferred (containing high amount of sugar and calories) compared to the locally made dishes which are very nutritious and healthy. Traditional diet are now been replaced with processed foods (67) because of their convenient nature. This is consistent with a recent study which was conducted in Zambia which linked living in urbanized areas having the potential of increasing obesity and weight (68). Also, urbanization has led to a better road network and availability of contemporary modes of transportation which has made people depend more on cars and commute less. People prefer boarding public transport to exercising by walking short distances, making it difficult for them to burn off the calories consumed, causing obesity. In several African countries despite the widespread poverty, access by the urban poor to cheap meals with high-fat content and sucrose content is easier (67, 69). In line with the study findings are the work of Pirgon and Aslan (44) where authors found out modern structural plans in urban areas families in urban locations do not send their children outside since there are no adequate walking and play places, so parents instead prefer to keep them home playing on a computer or watching television. After a certain age, the youngster develops by getting accustomed to a sedentary lifestyle and finds it difficult to socialize because of it. When it comes to urban planning, a lack of suitable locations for walking and cycling has been linked to obesity. However, negative relationship between urbanization and obesity in upper middle income and low middle-income countries was found in this study. Thus, urbanization is witnessed to contribute to the reduction in obesity in upper middle income and low middle-income countries. This can be as a result of urbanization making people more conscious and concerned of their health compared to those staying in less developed areas. Consistent with this study, Wang et al. (70) reported that the disparity could be owing to their greater access to health-related information. Thus, those staying in urbanized prefer selecting foods which have low calories compared to their counterparts (those living in less urbanized areas) with less health-related knowledge going for foods high in calories. Urbanization has led to creation of sport centers like gyms which are closer citizens and they walk short distances to keep fit or healthy at such places. For instance, in China groups of women meet in the community parks to dance in the form of exercise in urbanized areas.

Finally, the results revealed a strong positive relationship between trade and obesity among low-income countries where as a negative relationship in the aggregated panel, upper middle income and low middle income was found. This negative relationship implies that maybe most African countries are making prices of locally produced food products affordable and cheaper compared to imported food products. Also, domestic food products are likely to be less calorific and nutritional than imported foods, which reduces over nutrition. Further, to improve the quality of fats/oils and reduce sugars in the national food supply and combat the health risk associated with imported foods it is possible governments have utilized tariffs, import limits, or the withdrawal of subsidies for sugar and oil producers. This confirms the study conducted by Mary and Stoler (71) which found that a 1% increase in agricultural trade openness would lead to a decline in the prevalence of obesity by 0.5%. In the case of low-income countries, there is a positive relationship between obesity and trade. This suggests that low-income countries are allowing the influx of imported goods into their countries. For instance, most low-income countries signed a trade agreement termed the African Continental Free Trade Area (AfCFTA) and its promise to diversify economies, reduce reliance on exported goods, and boost regional trade. However, most countries still rely heavily on imported goods from Western countries and do not consider the health impact it has on their citizens. Fox et al. (2) reported that previous trade agreements signed between low-income countries and Western countries have increased people’s interest in the Western diet, which has shaped individuals’ tastes and preferences and increased the demand for obesogenic food products. In addition, increased access to developing countries’ markets for transnational food businesses to offer highly processed food, fast food, and other unhealthy commodities is generally associated with trade openness (72). To further support our study findings (19, 73) also reported in their studies a positive relationship between obesity and trade, and its influence on concentration in developing countries of which low-income countries are not an exception.

This study investigated the non-linear relationship between obesity and economic growth based on the Obesity Kuznet Curve framework. Although this current research has tempted to utilize as many observations as possible and applied robust econometric approaches in achieving the study’s objective, some limitations are worth mentioning. Most importantly, this current study focused on a panel framework and did not consider individual countries within the panel. Nonetheless, suppose respective countries are taken into consideration. In that case, such research will be more interesting since more diverse findings can be achieved and aid policymakers and other relevant parties in designing and implementing effective county-specific policies related to economic indicators-environment linkage. Also, in the case of examining the spatial pattern across African countries, the study only descriptively focused on the prevalence of Obesity among the employed African nations without taking to account the dynamics of the mentioned variable over time. Thus, this extent study recommends that, in future research, the dynamics of obesity over a well-defined period should be further reinvestigated using spatial–temporal analysis. Finally, relying on methods employed, this extant study considered one econometric model (Augmented Mean Group estimator) to estimate the non-linear relationship between economic growth and obesity. Nonetheless, the research could have benefited from a brief discussion of alternative econometric models that could be used to analyze the relationship between economic growth and obesity, such as instrumental variable regression or difference-in-differences estimation. This would provide readers with a better sense of the strengths and weaknesses of the chosen approach.

## 5. Conclusion and policy suggestions

Obesity has emerged as the world’s most pressing public health issue, with an increased risk of type 2 diabetes, stroke, myocardial infarction, and a variety of malignancies. This study employed a longitudinal panel data set from 1990 to 2020 from 38 Sub-Saharan African countries. The study found that unemployment, trade openness and urbanization was strongly associated with obesity in low-income countries. Furthermore, the study revealed that as the economy of a country develops, the relationship between income and obesity shifts over time. Thus, average obesity rates tend to rise and then fall after reaching a specific development threshold which is defined as the obesity Kuznets curve which is evident in the 38 Sub-Saharan African countries.

The empirical findings from this study are an indication that policymakers should closely monitor the high prevalence of obesity to minimize the negative effects on the population’s health. Based on the study findings the following recommendations are made;

1. First and foremost, government can introduce taxes on junk food, sugar-sweetened beverages or energy-dense foods with low nutritional value. These policies have been proven to be effective in some countries like Canada, the USA, Denmark, Finland, Hungry, Norway and Mexico (74), however, even though they did not completely eradicate the increase in obesity they aided in improving healthy eating habits. For instance, according to a study conducted by (74) a junk-food tax influences customers’ intertemporal choices through budget constraints and calorie awareness. This, as a result, competitive income, intertemporal substitution, and calorie-consciousness effects play a role in the tax’s influence on the body weight of the consumer. It is still clear that most developing countries of which Africa is not an exception still rely on foreign imported foods. Various African governments can introduce taxes to discourage the import of obesogenic foods and also promote domestic consumption of foods that are less caloric and healthy for citizens. A continuous effort from all African governments would make citizens have less taste for imported foods which are mostly junk foods and it can be possible if domestic healthy foods are made available and affordable to all.
2. Furthermore, in order to prevent and control obesity, African governmental policies should be tailored to the stage of the economic cycle, with a particular focus on the long-term unemployed. African nations should adopt measures to encourage healthy lifestyles which should focus on sedentary, long-term unemployed people with lower education levels. As a result, rather than a long-term health strategy, a shorter-term policy tailored to the specific phase of the economic cycle might be more successful. Further health programs and initiatives can be introduced to focus on health literacy understanding in schools, communities and media channels and also include new social media platforms (Facebook, WhatsApp, etc.) which are mostly used by citizens as a key setting for promoting healthy food and physical activity. Health programs should be inclusive for all thus poor obese people or unemployed individuals should be given priority in such health programs. Individuals alternatively, can also limit energy-dense, cook more at home instead of ordering takeout or delivery, take frequent breaks for short bouts of physical activity, schedule structured exercise sessions that can be done at home, establish daily routines, and find different outlets for stress such as meditation, yoga and exercises which have been proven to help in reducing obesity as cited by Zachary et al. (75). Creating public awareness of the importance of self-weighing regularly or daily (DSW). DSW has been demonstrated to be useful for weight loss (76), weight maintenance, and preventing age-related weight gain. Assuming people have access to a bathroom scale, it’s a reasonably cheap and simple instrument that can be used to try to avoid or limit shelter-in-place weight gain.
3. Policymakers and stakeholders in African countries may need to pay attention to the negative health externalities such as obesity, caused by urbanization, and figure out how to effectively address the problem. African policymakers should ensure there is equal distribution of development in both rural and urban areas. Also, healthy infrastructure for active transportation, environmental protection, and access to healthy and less expensive food supplies are all essential for long-term economic growth. Promoting national campaigns to ensure that development plans create safe pedestrian walkways and facilities for physical activities to enable citizens to feel safe going for walks and perhaps cycling.
4. Lastly, the study recommends policymakers to devise policies or initiatives to ensure that country’s economy grows at a steady pace. In this regard, the government should place a priority on implementing suitable health policies, including obesity prevention initiatives, to address the obesity problem. Also, a lancet report (77) has suggested that countries should have a paradigm shift from dominant development that is geared towards economic growth to a paradigm of sustainable development. The practical difficulty of sustainable development is figuring out how to achieve economic growth without compromising our natural environment or our health and well-being. For countries to be able to have sustainable development, it will necessitate policymakers to employ a coordinated policy-driven effort on a number of aspects of the current socioeconomic system (78).

## Data availability statement

The datasets presented in this study can be found in online repositories. The names of the repository/repositories and accession number(s) can be found at: https://data.worldbank.org/ and https://www.who.int/data.

## Author contributions

SA and IM: conceptualization, data curation, formal analysis, and roles/writing – original draft. HC: funding acquisition and supervision. HC, AY, and BD: investigation. SA, IM, and AY: methodology. IM: software. HC, AY, AT, and FA: validation and visualization. AY, BD, AT, and FA: writing – review and editing. All authors contributed to the article and approved the submitted version.
